# Assisted Reproductive Techniques in Latin America: The Latin American Registry, 2015

**DOI:** 10.5935/1518-0557.20190021

**Published:** 2019

**Authors:** Fernando Zegers-Hochschild, Juan Enrique Schwarze, Javier Crosby, Carolina Musri, Maria Teresa Urbina

**Affiliations:** 1 Unit of Reproductive Medicine Clínica Las Condes, Santiago, Chile; 2 Program of Ethics and Public Policies in Human Reproduction, University Diego Portales, Santiago, Chile; 3 Latin American Network of Assisted Reproduction (REDLARA), Montevideo, Uruguay; 4 Unifertes, Caracas, Venezuela

**Keywords:** ART, assisted reproductive technologies, multiple pregnancy, outcome, registry

## Abstract

**Research question:**

What was the utilization, effectiveness and safety of assisted reproductive technologies (ART) performed in Latin American countries during 2015, and what were the regional trends?

**Design:**

Retrospective collection of multinational data on assisted reproduction techniques (IVF and intracytoplasmic sperm injection [ICSI], frozen embryo transfer, oocyte donation, preimplantation genetic testing and fertility preservation), from 175 institutions in 15 Latin American countries.

**Results:**

In total, 41.25% of IVF/ICSI cycles were performed in women aged 35-39 years, and 28.35% in women aged ≥40 years. After removing freeze-all cycles, delivery rate per oocyte retrieval was 21.39% for ICSI and 24.29% for IVF. Multiple births included 19.58% twins and 0.95% triplets and higher. In oocyte donation, delivery rate per transfer was 36.77%, with a twin and triplet rate of 27.65% and 1.06%, respectively. Overall, preterm deliveries reached 17.38% in singletons, 64.94% in twins and 98.41% in triplets. Perinatal mortality in 14,936 births and 18,391 babies born was 10.5 per 1000 in singletons, 17.9 per 1000 in twins, and 57.1 per 1000 in high-order multiples. Elective single embryo transfer represented 3.11% of fresh transfers, with a 31.78% delivery rate per transfer. Elective double embryo transfer represented 23.3% of transfers, with a 37.79% delivery rate per transfer. Out of 18,391 babies born, 63.22% were singletons, 34.4% twins, and 2.38% triplets and higher.

**Conclusions:**

Given the effect of multiple births on prematurity, morbidity and perinatal mortality, reinforcing the existing trend of reducing the number of embryos transferred remains mandatory.

## INTRODUCTION

The Latin American Registry of Assisted Reproduction (RLA) was established  in 1990 as the first multinational and regional registry of assisted reproductive technology (ART). An annual report has been provided containing outcomes of ART procedures performed by institutions in most countries in Latin America, from Mexico in the north  to Chile in the south. Since 2010, individualized cycle-based data have been collected, thus establishing the first cycle- based multinational registry.

Over the years, the main objective of the RLA has been to disseminate information on ART procedures performed in  Latin America; this often serves as an external quality control to be used by institutions performing ART in the region and for other regions of the world. The regional database is also used to monitor outcomes, as well as trends in safety and efficacy, which contributes to developing better health interventions and appropriate public policies. Having access to an objective and external database is often well received by infertile couples when deciding if, when and what type of treatment should be undertaken. The RLA database is also used for epidemiological studies.

This report corresponds to the 27^th^ edition of the RLA. Previous reports, from 1990 to 1998, are available as printed copies; from 1999 to 2009 they are available as PDF files, which can be downloaded (www.redlara.com). Today, reports are published simultaneously in *Reproductive BioMedicine Online*, and in *JBRA Assisted Reproduction*, the official journal of REDLARA.

This report presents information on access/availability, effectiveness, safety and perinatal outcomes of ART treatment initiated between 1 January 2015 and  31 December 2015, and babies born up to September 2016.

## MATERIALS AND METHODS

Data on ART were collected from 175 centres in 15 countries in Latin America (Supplementary Table 1), covering  fresh autologous cycles of IVF and intracytoplasmic sperm injection (ICSI); frozen embryo transfer (FET); oocyte donation (OD) including the transfer of both fresh and frozen-thawed embryos; fertility preservation (FP); and oocyte cryopreservation cycles, both autologous and heterologous.

This report includes treatments started between 1 January 2015 and  31 December 2015. Data on pregnancy and neonatal outcomes are obtained from follow-up of the cohort treated during this period.

As part of the accreditation programme, all participating institutions agree to have their data registered and published by the RLA. Therefore, no other consent form was requested for the scientific disclosure of these data.

The method of collecting data in 2015 resembles previous years, making results comparable. Briefly, each institution enters their data directly into an online RLA web-based system, with built-in algorithms for internal consistency. Any error or discrepancy not identified by the software is discussed and clarified by RLA’s central office. Given that the RLA is a voluntary multinational registry, centres are not obliged to upload each case immediately the cycle is initiated. Therefore, some cases are sent to the RLA upon patient recruitment while others are included retrospectively. Given that there is no obligation to include each case upon recruitment, approximately 90% of cycles are reported retrospectively. This can affect overall results because there could be a selection of predominantly those initiated cycles that advanced towards aspiration.

Definitions used refer to the glossary developed by the International Committee for Monitoring Assisted Reproductive Technologies (ICMART) and the World Health Organization (WHO) (Zegers-Hochschild *et al.,* 2009). Preimplantation genetic diagnosis and screening are registered together as preimplantation genetic testing (PGT) (Zegers-Hochschild *et al.,* 2017a;b).

When calculating clinical pregnancy  or delivery rates per oocyte pick-up, cases of total embryo freezing were not included in the calculation.

Cumulative live birth rate by age category was calculated for autologous cycles performed between 2012 and 2015. Each patient was identified by  a personal identification number and date of birth, and her embryo transfers were considered -both fresh and frozen-thawed - until one of two things occurred: a delivery or the transfer of all embryos generated in the corresponding oocyte pick-up. The identification number is not yet universal in Latin America, so not all patients could be followed and it is also possible that cross-border reproductive treatments could partially influence results, but the numbers should be small. Furthermore, it was not possible to follow up individual patients in all reporting institutions; only those in which a consistent ID number was used throughout the study period could be followed up.

In order to test for the effect of age on delivery rate per embryo transfer, logistic regression analysis was performed,  in both fresh and OD cycles. When appropriate, a chi-squared test was used to analyse independence of categorical variables. A *p*-value less than 0.05 was considered statistically significant.

## RESULTS

### Participation

One hundred and seventy-five centres in 15 countries reported ART procedures performed during 2015. This represents approximately 70% of centres in the region. Most centres were in Brazil (n=58), followed by Mexico (n=31) and Argentina (n=29) (Table 1).

**Table 1:** Assisted Reproduction Technique procedures reported to RLA and access in 2015

	Centres	FP	FRESH	FET	OD	Other	Total	Access(a)
Argentina	29	655	10003	3638	3247	245	17788	409
Bolivia	3	2	483	47	151	5	688	64
Brazil	58	1510	18058	8407	2255	986	31216	153
Chile	10	268	2262	1101	644	194	4469	255
Colombia	11	28	1101	259	431	38	1857	40
Dominican Rep.	2	0	162	8	89	0	259	25
Ecuador	5	4	328	106	149	3	590	37
Guatemala	1	4	119	36	31	0	190	13
Mexico	31	164	5433	1746	2780	179	10302	85
Nicaragua	1	0	131	0	24	0	155	26
Panama	3	17	482	142	118	24	783	214
Paraguay	1	3	87	24	15	4	133	20
Peru	11	531	1835	622	1353	459	4800	158
Uruguay	2	13	335	80	78	4	510	153
Venezuela	7	33	828	193	322	5	1381	45
Total	175	3232	41647	16409	11687	2146	75121	133

FET=initiated frozen autologous embryo; FP=fertility preservation; FRESH=initiated IVF/ICSI cycles; OD=initiated cycles for transfer of fresh or frozen embryos using donated oocytes; Other=the transfer of embryos derived from froze–/thawed autologous and donated oocytes.

(a) Number of initiated cycles in the country per million population in 2015 (World Population Data Sheet, World Bank).

### Size of participating institutions

A total of 75,121 initiated cycles were reported (14.6% more than the previous year), corresponding to the sum of IVF/ ICSI, FET, OD and FP. Cycles of embryos transferred after frozen-thawed oocytes, either own or donated, were grouped as OTHER.

The mean number of initiated cycles by institution was 429, with wide variation; 19% performed ≤100 cycles; 33% between 101 and 250 cycles; 23% between 251 and 500 cycles; 15% between 501 and 1000 cycles; and 10% >1000 cycles.

### Number of treatment cycles per technique and availability 

Out of 75,121 initiated cycles, 41,647 corresponded to IVF/ICSI (representing 9.3% increase over 2014); 16,409 FET (21.1% increase); 11,687 OD (4.4% increase), 3232 FP (19.3% more cycles than 2014), and 2146 cycles reported as OTHER.

Of the 41,647 initiated IVF/ICSI  cycles, at least one mature oocyte was recovered in 38,448 aspirations (92.3% of cases). The preferred method  for insemination was ICSI (85.5%)  and, including both IVF and ICSI, at least one embryo was transferred  in 25,554 cases. The main reasons  for no embryo transfer were: 8802 cases of total embryo freezing, 2058 cases of abnormal in-vitro embryo development, and 1192 cases of total fertilization failure corresponding to 3.1% of inseminations. There were 836 cases where no normal embryos were obtained after PGT. There were only six cases where the reason for no embryo transfer was unknown.

Utilization of ART is still very low in Latin America; in 2015 it reached 133 initiated cycles per million people, ranging from 13 cycles per million in Guatemala to 409 cycles per million in Argentina (Table 1). It is important to mention that not all centres performing ART report to the RLA. It is estimated that overall, 75% of centres report, including the majority of institutions performing ≥1000 cycles per year.

### Outcome of pregnancies and deliveries

In the present year, 19,601 clinical pregnancies were reported, of which 983 (5%) were lost to follow-up. Thus, the analysis of outcome variables should not be affected by these losses. Table 2 shows the clinical pregnancy rate (CPR) and delivery rate (DR) per oocyte pick- up (OPU) in IVF/ICSI cycles. Both CPR and DR per OPU were higher in IVF cycles than in ICSI cycles (31.49% and 28.62%, *p*<0.001; 24.29% and 21.39%, *p*<0.001, respectively). Furthermore, as shown previously, both CPR and DR per ET were much higher in OD than in autologous reproduction, reaching 45.97% and 36.77%, respectively. Also, in FET cycles, CPR and DR per transfer were 36.79% and 27.81%, respectively (Table 3).

**Table 2:** Clinical pregnancy rate and delivery rate in IVF and intracytoplasmic sperm injection cycles in 2015

Assisted reproduction technique procedure	Oocyte retrieval^a^	Clinical pregnancy rate per oocyte retrieval (%)	Delivery rate per oocyte retrieval (%)
**ICSI**	25,599	28.62	21.39
**IVF**	4,417	31.49	24.29
***p*-value**	----	<0.001	<0.001

a ocyte retrieval with at least one mature oocyte

**Table 3:** Clinical pregnancy rate and delivery rate by embryo transfer in oocyte donation and FET cycles in 2015

Assisted reproduction technique procedure	Embryo transfer	Clinical pregnancy per embryo transfer (%)	Delivery rate per embryo transfer (%)
**Oocyte Donation**	9503	45.97	36.77
**Frozen-thawedembryo transfer**	15,844	36.79	27.81

### Age distribution

**Figure 1:**
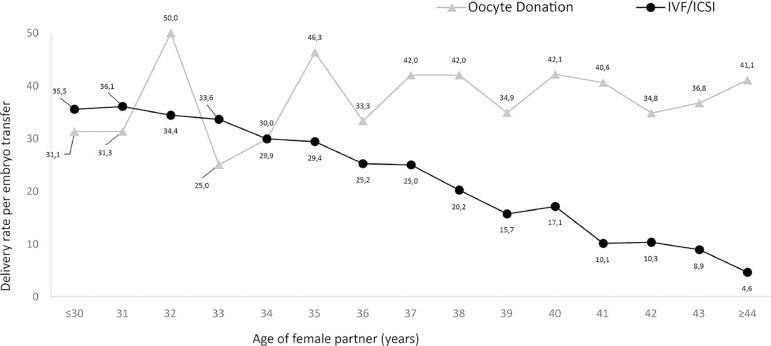
Delivery rate per embryo transfer in IVF/intracytoplasmic sperm injection (ICSI) and oocyte donation cycles (RLA 2015)

The mean age of women undergoing IVF/ICSI was 36.2 years (SD 4.6). The majority of cycles were performed in women aged 35 to 39 years (41.25%), followed by 28.35% of women aged ≥40 years, meaning that 69.6% of women using autologous ART were ≥35 years. The mean age of women undergoing fresh OD was 41.0 (SD 5.3); and the majority of cycles (40.97%) were performed in women aged ≥42 years. As expected, the DR per embryo transfer decreased with advancing age in the case of autologous IVF/ICSI, but not in OD (Figure 1).

### Number of embryos transferred and multiple births

Table 4 summarizes the number of embryos transferred and multiple births after IVF/ICSI, with a mean of 2.02 embryos (range 1 to 6). There were 5069 single embryo transfers (SET), which correspond to 19.84% of all transfers.  Of these, only 796 were elective (eSET), representing 3.11% of ET. There were 15,560 double embryo transfers (DET), which correspond to 60.90% of ET,  of which 5954 (23.30% of all ET) were elective (eDET).

**Table 4:** Clinical pregnancy rate, delivery rate and gestational order according to the number of embryos transferred in IVF and intracytoplasmic sperm injection cycles in 2015

Number of transferred embryos	Total embryo transfer		Clinical pregnancy rate per embryo transfer (%)	Deliveries				
	Number	%		Total (number)	Delivery rate per embryo transfer (%)	Singleton (%)	Twin (%)	≥ Triplets (%)
1	5,069	19.84	19.92	747	14.74	97.46	2.54	0.00
2	15,560	60.90	37.91	4,493	28.88	77.92	21.79	0.29
3	4,395	17.20	37.05	1,197	27.24	74.69	21.72	3.59
≥4	530	2.07	31.99	111	20.94	72.97	21.62	5.41
Total	25,554	100.00	34.07	6,548	25.62	79.47	19.58	0.95

Overall, the CPR and DR per ET reached 34.07% and 25.62%, respectively, in cases of eSET, and the DR per ET reached 31.78%, increasing to 37.79% in eDET. In terms of multiple births, of the 6548 IVF/ICSI deliveries registered, 79.47% were singletons, 19.58% were twins, and 0.95% were triplets.

### Number of embryos transferred after IVF/ICSI according to the age of women 

In women ≤34 years, the mean number of embryos transferred was 1.98 (range 1 to 5). In this age group, 15.02% were SET and 4.8% eSET, 71.95% DET and 34.3% eDET, and 12.96% TET (three embryos transferred) including very few cases (0.7%) with four or more embryos transferred.

In women between 35 and 39 years, the mean number of embryos transferred was 2.02 (range 1 to 6). In this age group, 19.6% were SET and 3.1% eSET; 60.3% DET and 22.9% eDET, and 18.9% TET; while the transfer of four or more embryos occurred in 1.2% of transfers.

In women ≥40 years of age, the mean number of embryos transferred was 2.05 (range 1 to 6). In this age group, 25.9% were SET and 1.0% eSET, 48.6% DET and 10.5% eDET, and 20.4% TET; while the transfer of four or more embryos occurred in 5.1% of transfers.

### Number of embryos transferred and multiple births after OD and FET

Table 5 summarizes the number of embryo transfers and multiple births in OD (fresh and FET), where the mean number of embryos transferred reached 2.01 (range 1 to 5). There were 1624 SET, which correspond to 17.09% of ET and 399 were eSET, representing 4.20% of all ET/OD. There were 6226 DET, which correspond to 65.52% of ET, and 2200 were eDET, representing 23.15% of all ET/OD.

**Table 5:** Clinical pregnancy rate, delivery rate and gestational order according to the number of embryos transferred in fresh and cryopreserved oocyte donation cycles in 2015

Number of transferred embryos	Total embryo transfer		Clinical pregnancy rate per embryo transfer (%)	Deliveries				
	Number	%		Total (number)	Delivery rate per embryo transfer (%)	Singleton (%)	Twin (%)	≥Triplets (%)
1	1,624	17.09	36.76	442	27.22	97.74	2.26	0.00
2	6,226	65.52	47.64	2,374	38.13	68.53	30.92	0.55
3	1,563	16.45	49.46	650	41.59	62.77	33.54	3.69
≥4	90	0.95	36.67	28	31.11	85.71	14.29	0.00
Total	9,503	100.00	45.97	3,494	36.77	71.29	27.65	1.06

Overall, the CPR and DR per ET were 45.97% and 36.77%, respectively. Of the 3494 deliveries registered, 71.29% were singletons, 27.65% were twins and 1.06% were triplets and higher. Furthermore, DR/ET was not affected by the age of the oocyte recipient (OR 0.99, 95% CI 0.97-1.02) (Figure 1).

Table 6 summarizes the number of embryos transferred in FET, where the mean number of embryos transferred reached 1.87 (range 1 to 6). There were 4112 SET, which correspond to 25.95% of ET. There were 9852 DET, which correspond to 62.18% of ET. Overall the CPR and DR per ET reached 36.79% and 27.81%, respectively. Of the 4407 deliveries registered, 80.94% were singletons, 18.11% were twins, and 0.95% were triplets and higher.

**Table 6:** Clinical pregnancy rate, delivery rate and gestational order according to the number of embryos transferred in frozen embryo transfer cycles in 2015

Number of transferred embryos	Total embryo transfer		Clinical pregnancy rate per embryo transfer (%)	Deliveries				
	Number	%		Total (number)	Delivery rate per embryo transfer (%)	Singleton (%)	Twin (%)	≥Triplets (%)
1	4,112	25.95	30.91	927	22.54	98.38	1.62	0.00
2	9,852	62.18	39.28	2,964	30.09	76.55	22.64	0.81
3	1,778	11.22	36.95	500	28.12	75.00	21.40	3.60
≥4	102	0.64	30.39	16	15.69	68.75	31.25	0.00
Total	15,844	100.00	36.79	4,407	27.81	80.94	18.11	0.95

### Influence of stage of embryo development at transfer

Overall, 42.20% of ET were performed at the blastocyst stage. In fresh IVF/ ICSI, OD and FET cycles the rates of blastocyst transfer were 28.56%, 54.92% and 54.98%, respectively.

Blastocyst transfers were always associated with an increase in the DR/ET compared with cleavage-stage embryos, irrespective of whether fresh  or frozen and the number of embryos transferred. In the case of IVF/ICSI, the DR/ET of blastocysts versus cleaving embryos was 32.74% and 22.75%, respectively (*p*<0.0001). In OD, the DR/ET was 42.10% and 30.28%, respectively (*p*<0.0001); and in FET, the DR/ET was 32.83% and 21.69% (*p*<0.0001).

### Perinatal outcome and complications

Table 7 summarizes perinatal mortality. Data were available from 14,936 births and 18,391 babies born. The perinatal mortality increased from 10.5 per 1000 births in 11,627 singletons, to 17.9 per 1000 in 6326 twins and 57.1 per 1000 in 438 triplets and higher. Overall, 36.8% of babies were born multiples. In the case of fresh OD, this proportion increased  to 45.1%, while in the case of IVF/ICSI in women younger than 35, the proportion of multiple babies reached 28.29% of the 673 newborns.

**Table 7:** Perinatal mortality according to gestational order in 2015

Assisted reproduction technique procedure	Singleton			Twin			≥Triplets		
	Live birth	Still birth	Early neonatal death	Live birth	Still birth	Early neonatal death	Live birth	Still birth	Early neonatal death
FET	3,542	11	14	1,579	2	15	117	2	7
Fresh	5,151	20	33	2,516	13	35	177	3	6
OD	2,450	16	25	1,892	22	18	104	3	4
Other	362	2	1	226	3	5	15	0	0
Total	11,505	49	73	6,213	40	73	413	8	17
Perinatal mortality^a^	10.5			17.9			57.1		

a Proportion of still births plus early neonatal death per 1,000 newborns FET= frozen embryo transfer; ICSI=intracytoplasmic sperm injection

Gestational age at delivery was reported in 12,906 deliveries (86.4%). The mean gestational age at delivery was 37.6 (SD 2.2) weeks in singletons, 35.2 (SD 2.7) weeks in twins, and 32.08 (SD 3.1) weeks in triplets and higher. The overall risk of preterm birth (gestational weeks 20-36) increased from 17.38% in singletons, to 64.94% in twins, and 98.41% in triplets and higher. Furthermore, the risk of very preterm birth (gestational weeks 20-27) increased from 0.79% in singleton to 2.43% in twins and to 8.73% in triplets and higher.

During 2015, 118 cases of severe ovarian hyperstimulation syndrome requiring hospitalization or major medical interventions were reported, together with 25 cases of haemorrhage, and four cases of infection presumably associated with ovarian puncture. It is likely that these conditions are under-reported and only the most severe cases are reported.

### Total embryo freezing

A total of 8802 cycles of total embryo freezing were reported, 36.4% more than in 2014. On average 4.3 embryos (SD 3.4) were cryopreserved. Out of these cases, 3600 cycles of FET were reported, with 1080 deliveries and the DR/ET was 30.0%, which is higher than a mean of 27.81% of DR/ET in FET cycles that follow fresh cycles (*p*=0.0092). A second FET attempt was reported in 977 cases from the same cohort, with 250 subsequent deliveries, the DR/ET was 25.59%. Adding all transfers from the total freeze cohort makes a 29.1% DR per ET.

### Preimplantation genetic testing (PGT)

The RLA registers PGT-M and PGT-A together. Ninety-seven centres reported these procedures in 2859 fresh cycles, 749 using frozen-thawed embryos and 313 in OD. The mean age of women undergoing PGT was 37.5 (SD 4.3) among fresh cycles, 37.4 (SD 4.5) in FET and 40.8 (SD 4.8) in OD.

In the case of fresh cycles, the mean number of embryos biopsied was 3.3 (SD 2.4), and the mean number of normal embryos was 1.1 (SD 1.5). In the case  of FET, the mean number of embryos biopsied was 3.2 (SD 2.5), and the mean number of normal embryos was 1.8  (SD 1.4). In the case of OD, the mean number of embryos biopsied was 4.7 (SD 2.7), and the mean number of normal embryos was 2.4 (SD 1.9).

The mean number of embryos transferred was 1.5 (SD 0.6) in fresh cycles, 1.4 (SD 0.5) in FET and 1.5 (SD 0.5) in OD. The miscarriage rate reached 21.2% in fresh, 13.4% in FET and 12.5% in OD. The DR/ ET was 21.89% in IVF/ICSI cycles, 32.48% in FET and 34.45% in OD.

### Fertility preservation (FP)

A total of 3232 initiated cycles for FP were reported in 2015. The mean age of women was 35.6 (SD 5.4) years, range 18 to 51 years. In 189 aspirations, no oocytes were available for cryopreservation. The mean number of oocytes cryopreserved was 7.5 (SD 6.2), range 1 to 54. In  cases where the indication for FP was recorded, the majority were related to the desire to postpone pregnancy (2213 cases), while cancer-related factors were reported in 237 cases; risk of premature ovarian insufficiency in 122 cases and other reasons in 660 cases.

### Cumulative delivery rate (CDR)

**Figure 2:**
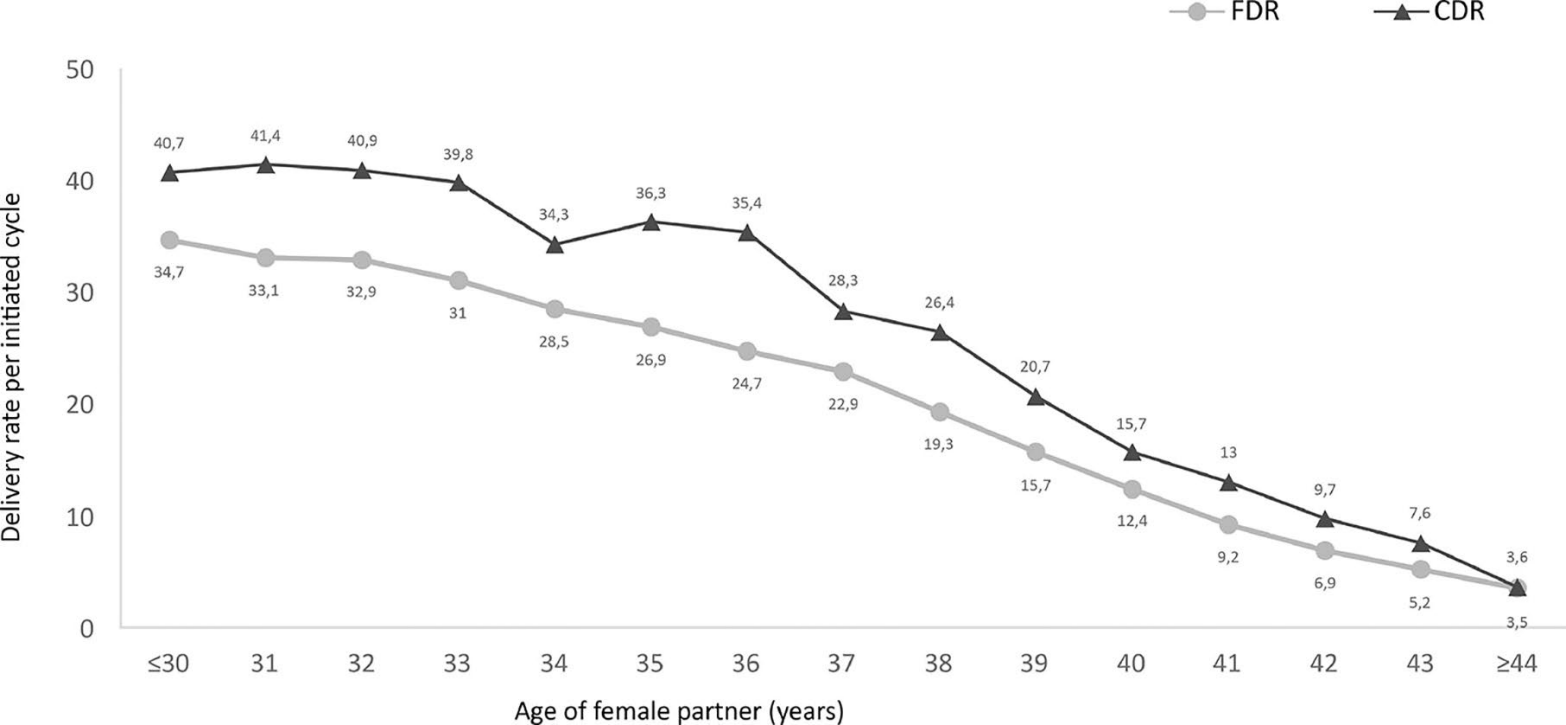
Cumulative delivery rate (CDR) and fresh delivery rate (FDR) per initiated cycle from 2012 to 2015, according to a woman's age

The cumulative delivery rate per transfer (CDR) of 14,424 patients treated between 2012 and 2015 are presented. The CDR per woman was estimated by considering the outcome of fresh embryos and all FET. Cases were censored once a delivery occurred or all embryos (both fresh and frozen- thawed) were transferred. Results are presented according to the age of the female partner. These data are compared with the DR per ET of fresh cycles of all women treated during that period (Figure 2).

## DISCUSSION

The present report is the 27^th^ consecutive annual RLA report on ART procedures performed in Latin America. It is estimated that more than 75% of the cycles performed in the region are presented.

Overall, the number of reported initiated cycles increased by 15% (Zegers-Hochschild *et al*., 2017a;b) with respect to the previous year. However, access to ART in Latin America (133 initiated cycles/million population) remains very much under the threshold of 1500 cycles per annum per million inhabitants proposed by the ESHRE Capri Group, in order to fulfil the ART needs of a population (The ESHRE Capri Workshop Group, 2001). It is worth mentioning that Argentina is the first country in Latin America to legislate in favour of universal access to infertility treatment (2013); correspondingly it is the country with the highest access to ART (409 cycles/million population) and increasing. This reproductive rights initiative was then followed by Uruguay and Costa Rica. It will take some time to appreciate its full impact in ART utilization.

The rise in the number of initiated cycles is mainly a result of an increase in FET. This increase in FET cycles is partly explained by a modest increase in the proportion of elective SET and DET, but mostly by an increase of 36.4% in the number of cycles with total embryo cryopreservation.

The reporting of efficacy of ART can be presented in different ways. Because  the number of freeze-all cycles has increased, the DR per OPU excluding freeze-all cycles is presented here, and the data are also presented as DR per ET. The overall DR per ET for fresh non- donor cycles (25.6%) is higher than that in the latest report by the EIM (23.4%), but lower than the latest report by the CDC (36.7%) (European IVF-monitoring Consortium (EIM); European Society of Human Reproduction and Embryology (ESHRE), 2017; U.S. Department of Health and Human Services, Centres for Disease Control and Prevention, 2017). As expected, this outcome is influenced by the stage of embryo development at transfer, number of embryos transferred and age of the female partner. The huge difference in outcome reported by Latin America and the USA results from differences  in the age of the population. While the median age of patients in the USA is 35, in Latin America it is 36. Furthermore, the proportion of women ≥40 years increased from 23% in the USA to  28% in Latin America. Similarly, the proportion of women under 30 years of age in the USA is 12% and only 6.6% in Latin America. These differences partly explain the overall better outcome in the USA compared with Latin America.

The effect of women's age on treatment outcome is well represented in Figure 1. The DR per ET in non-donor oocytes drops as age increases while it remained fairly stable when donor oocytes were used, regardless of the age of the recipient.

The mean number of embryos transferred in IVF/ICSI decreased  from 2.40 in 2010 to 2.02 in 2015. The proportion of DET (60.90%) is larger than that published by the EIM (56.3%) and by the CDC (52.3%). However, the proportion of SET (19.84%) is much lower than that reported by the EIM (31.4%) and by the CDC (33.5%) (European IVF-monitoring Consortium (EIM); European Society of Human Reproduction and Embryology (ESHRE), 2017; U.S. Department of Health and Human Services, Centres for Disease Control and Prevention, 2017).

It is very alarming that even in patients with good prognosis, e.g. patients under 35 years undergoing fresh IVF/ICSI or patients undergoing OD cycles, three  or more embryos were transferred in 13.1% and 17.4% of ET, respectively. The most plausible explanation for what might be considered a form of poor clinical practice relies on the fact that in Latin America most ART procedures are funded out-of-pocket. Therefore, both physicians and patients try to improve the outcome of any given cycle in its first attempt, transferring more embryos. This accounts for the high rate of multiple births, preterm and extreme preterm births and its perinatal consequences.

Indeed, in the case of non-donor fresh ET, the proportion of delivery of triplets reached 0.95%, higher than that reported by CDC (0.6%) and EIM (0.5%).  When donated oocytes were used, the proportion of delivery of triplets reached 1.06%, higher than that reported by CDC (0.4%) and EIM (0.4%) (European IVF-monitoring Consortium (EIM); European Society of Human Reproduction and Embryology (ESHRE), 2017; U.S. Department of Health and Human Services, Centres for Disease Control and Prevention, 2017). It is perhaps worth mentioning that selective embryo reduction is seldom performed in Latin America; while although not officially reported by CDC, it is a well-established practice in the USA.

Multiple deliveries, especially high-order, were associated with an increase in the risk of perinatal death, even in the case of twin deliveries. Therefore, multiple embryo transfer should be strongly discouraged.

To persuade both clinicians and patients  of the benefits of transferring fewer embryos, success should rely on the CDR. This report presents for the first time the cumulative live birth rate per initiated cycle. However, the frequently long temporal lag until all FET resulting from the same cycle are transferred creates obstacles to interpreting the data correctly. Also, the frequent geographic movements of people and cross-border reproductive care represent another issue to overcome. Nevertheless, it was possible to follow more than 14,000 fresh cycles followed by FET. In every age category, the CDR is significantly higher than the fresh transfer only. As expected, a woman’s age strongly affects both fresh transfer and the sum of the fresh + FET. In this cohort, the ceiling reached by young women does not exceed 40 to 41 deliveries per 100 initiated cycles, and there are minor differences among  the three main contributing countries. However individual centres, transferring only blastocysts, can reach cumulative birth rates of >70% in women aged 30-34 years.

Latin America is moving in the right direction and the education of both clinicians and patients towards reducing the number of embryos to transfer should be pursued, especially in patients with good prognosis.

## Supplementary material

**Table 1:** Centres reporting to Latin America Registry of ART in 2015

ARGENTINA
• Instituto de Fertilidad Asistida
• Centro de Estudios en Ginecología y Reproducción (CEGYR)
• Centro de Salud Reproductiva (CER)
• Instituto Tersoglio
• Centro Integral de Ginecología, Obstetricia y Reproducción (CIGOR)
• Centro de Investigaciones en Medicina Reproductiva (CIMER)
• Centro de Medicina Reproductiva Bariloche
• Centro de Estudios en Reproducción y Procedimientos de Fertilización Asistida (CRECER)
• FECUNDITAS
• FERTILAB
• GESTAR
• Centro de Reproducción Fertilequip
• Fertya
• Gens, Centro especializado en tratamientos para la mujer
• Hospital de Clínicas
• FECUNDART
• Instituto de Medicina Reproductiva
• Centro de Reproducción, servicio de Ginecología Hospital Italiano
• Mater, Medicina Reproductiva
• Nascentis, Medicina Reproductiva
• HALITUS, Instituto Médico
• Instituto Medico de ginecología y Fertilidad PREFER
• PREGNA, Medicina Reproductiva
• Programa de asistencia reproductiva PROAR
• PROCREARTE
• Fertilidad San Isidro
• SARESA, Salud reproductiva Salta
• SEREMAS
• VITAE, Medicina Reproductiva

BOLIVIA
• CENALFES
• Instituto de Salud Reproductiva (ISARE)
• EMBRIOVID, centro integral de reproducción y especialidades médicas

BRAZIL
• ANDROLAB, Clinica y Laboratorio de Reproducción Humana y Andrología
• ANDROFERT, Centro de Referencia en Reproducción Masculina
• FERTIVITRO, Centro de Reproducción Humana
• BIOS, Centro de Medicina Reproductiva
• FIV-MED
• Centro de Medicina Reproductiva
• VIDA, Centro de Fertilidad REDE D’OR
• Clinica FERTWAY
• NASCER, medicina reproductiva ltda.
• ORIGINARE, Centro de Investigación y Reproducción Humana
• CLINIFERT, Centro de Reproducción Humana
• CONCEPTUS, Centro de Reproducción Asistida de Cear
• CONCEBER, Centro de Medicina Reproductiva
• Clinica Pro-Genesis
• Centro de reproducción humana CONCEPTION
• Centro de Reproducción Humana MONTELEONE
• Fértile Diagnósticos
• CEERH, Centro especializado en Reproducción Humana
• Embrios, centro de reproducción humana
• EMBRYOLIFE, Instituto de Medicina Reproductiva
• Centro de Reproducción Humana, Endoscopia y Medicina Fetal de Bahía (CENAFERT)
• Instituto VERHUM
• Clinica FERTIBABY BH
• FECUNDA, Reproducción Humana
• FELICCITA, Instituto de Fertilidad Ltda.
• HUMANA, Medicina Reproductiva (Ex centro de Reproducción asistida FEMINA)
• FERTILITY, Centro de Fertilización Asistida de Campo Grande
• FERTILITY, Centro de Fertilización Asistida
• FERTIL Reproduccion Humana
• REPROFERTY
• FERTICLIN, Clínica de Fertilidad Humana
• GENESIS, Centro de Asistencia en Reproducción Humana
• Clinica Genics, medicina reproductiva y genómica
• FERTIPRAXIS, Centro de Reproducción Humana (Ex Fert. Gin. y Obst. de Barra)
• GERA, Grupo de endoscopia y Reproducción Asistida
• Clinica GERAR VIDA
• Instituto de Saude Da Mulher, Cegonha Medicina Reproductiva
• IVI Sao Paulo, Chedid Grieco S.A.
• HUMANA (PRIMORDIA, Medicina Reproductiva Huntington RJ)
• Hospital de Clínicas de Riberao Preto
• HUNTINGTON Campinas
• HUNTINGTON, Centro de Medicina Reproductiva (Sao Paulo)
• JULES WHITE, Centro de Medicina Reproductiva
• HUNTINGTON Vila Maria
• IMR, Instituto de Medicina Reproductiva e Fetal
• Servicio de Reproducción Humana Del Hospital y Maternidad Santa Johana
• Life reproducción humana
• FERTILITAT, Centro de Medicina Reproductiva
• Clínica MATRIX
• Pro-criar Monte Sinaí
• Centro de Reproducción Humana Nilo Frantz
• Clínica ORIGEN
• Clínica PRO-CRIAR, Medicina Reproductiva
• Clínica PRO NASCER
• Centro de Reproducción Humana De San Jose de Rio Preto
• GENESIS, Centro de Reproducción Humana
• Centro de Reproducción Humana Prof. Franco Junior
• Centro de Ensino y Pesquisa en Reproducción Asistida (Centro de Rep. Asist. Hospital Da ASA SUL)

CHILE
• UMR Clínica de la Mujer Antofagasta
• Centro de Estudios Reproductivos (CER)
• Unidad de Medicina Reproductiva, Clínica Alemana
• Unidad de Medicina Reproductiva, Clínica las Condes
• Unidad de Medicina Reproductiva, Clínica de la Mujer
• IVI Santiago de Chile
• Programa e Fertilización Asistida I.D.I.M.I.
• Clínica Monteblanco
• Centro de Fertilidad y Medicina Reproductiva Concepción S.A.
• Centro de reproducción humana

COLOMBIA
• Centro FECUNDAR, Cali
• Unidad de fertilidad del Coutry ltda. CONCEPTUM
• Asociados en Fertilidad y Reproducción Humana
• FERTIVIDA
• Clinica Machicado SAS
• Centro Médico IMBANACO
• Instituto de Fertilidad Humana S.A.S. (INSER)
• IN SER, Instituto Antioqueño de Reproducción
• Profamilia Fertil
• Unidad de Fertilidad, Procreación Medicamente Asistida
• Union temporal IN SER eje cafetero

ECUADOR
• Clínica de Medicina Reproductiva BIOGEPA
• Clínica INFES
• Instituto Nacional de Investigación de Fertilidad y Esterilidad (INNAIFEST)
• CONCEBIR, Unidad de Fertilidad y Esterilidad
• Unidad de Fertilidad Hospital Alcívar

GUATEMALA
• Centro de Reproducción Humana S.A. (CER)

MEXICO
• Biofertility Center
• Centro de Diagnóstico Ginecológico
• URA, Unidad de reproducción asistida de Hispital CIMA Hermosillo
• Instituto para el estudio de la Concepción Humana IECH
• Centro de Reproducción Asistida del Hospital Español (HISPAREP)
• Centro de Reproducción Asistida del Occidente
• Centro de Reproducción Asistida de Saltillo
• Centro Universitario de Medicina Reproductiva
• CREASIS SC
• Fertility Center Cancún
• Ginecología y Reproducción Asistida GYRA
• Grupo de reproducción y genética AGN y asociados
• Instituto para el estudio de la concepción humana de Baja California
• Instituto Mexicano de Alta Tecnología Reproductiva S.C. (INMATER)
• Instituto de medicina reproductiva del Bajío IMER, sede Guadalajara
• Instituto IMER de Tijuana
• Instituto Mexicano de infertilidad
• Instituto Médico de la mujer (RED CREA)
• Instituto de Ciencias en Reproducción Humana, sede Guadalajara
• Instituto de Ciencias en Reproducción Humana, sede Matamoros
• Centro especializado para la atención de la mujer (CEPAM)
• INGENES
• INGENES Guadalajara
• Instituto de Ciencias en Reproducción Humana (VIDA), sede León
• Medica Fertil
• Instituto de ciencias en reproducción humana del Sureste (Vida Merida)
• Centro de Medicina Reproductiva FILIUS
• PROGEN, Reproducción asistida y medicina fetal
• Clinica de Infertilidad y reproducción asistida de Toluca SA de CV
• Centro especializado en esterilidad y Reproducción Humana (CEERH)
• Instituto de Ciencias en reproducción humana VIDA, ciudad de Mexico.

• NICARAGUA
• Centro de Fertilidad de Nicaragua

PANAMA
• IVI Panamá S.A.
• Centro de reproducción Punta Pacífica
• Instituto de salud femenina

PARAGUAY
• Neolife, Medicina y cirugía reproductiva

PERU
• Clínica CEFRA, Centro de Fertilidad y Reproducción Asistida
• CERFEGIN
• Centro de Fertilidad y Ginecología del Sur (CFGS)
• Clinica de fertilidad del norte, Clinifer de Chiclayo
• FERTILAB, Laboratorio de Reproducción asistida
• Inmater, Clinica de fertilidad
• Clínica Miraflores, Instituto de Ginecología y Fertilidad
• Nacer
• Grupo Pranor, Clínica CONCEBIR
• Grupo Pranor, Instituto de Ginecología y Reproducción
• Pranor, laboratorio de medicina reproductiva sede trujillo

REPUBLICA DOMINICANA
• Instituto de Reproducción y Ginecología del CIBAO (IREGCI)
• PROFERT

URUGUAY
• Centro de Esterilidad Montevideo (CEM)
• Centro de Reproducción Humana del Interior

VENEZUELA
• FERTILAB
• UNIFERTES
• Centro Medico docente la Trinidad
• EMBRIOS, Centro de Fertilidad y Reproducción Humana, Hospital de Clínicas de Caracas
• GENESIS, Unidad de Fertilidad y Reproducción
• Instituto Venezolano de Fertilidad
• Laboratorios In Vitro de Venezuela
